# Facial Emotion Recognition in Sleep Deprivation: A Systematic Review and Meta-Analysis

**DOI:** 10.5334/irsp.679

**Published:** 2023-06-12

**Authors:** Mengyuan Li, Chifen Ma, Chao Wu

**Affiliations:** 1 Peking University School of Nursing, 38 Xueyuan Road, Haidian District, Beijing 100191, China; 2 College of Health Services and Management, Xuzhou Kindergarten Teachers College, Xuzhou 221001, China

**Keywords:** sleep deprivation, insomnia, emotion processing, facial emotional recognition, meta-analysis

## Abstract

**Objectives:**

Sleep deprivation (SD) has detrimental effects on cognition. Emotional processing, a critical component of social cognition, is also affected by SD. However, current research on how SD affects emotion recognition and the specific emotion recognition that declines with SD is inconsistent. The present study meta-analyzed results of studies examining emotion category recognition changes in SD compared with controls.

**Methods:**

PubMed, Web of Science, Embase, PsychINFO, MEDLINE, and China National Knowledge Infrastructure databases were searched. Studies on the impact of acute SD or insomnia on emotional recognition and participants aged 18 years or older were included in this review. The JBI Critical Appraisal Checklist and GRADE approach were used to assess the quality of the studies and evidence.

**Results:**

Twelve studies with 414 SD and 399 control participants were included in this meta-analysis. The SD group performed poorer on facial sadness (MD = –4.35; 95% CI, –7.99 to –0.71) and happiness (MD = –1.75; 95% CI, –3.25 to –0.26) recognition than the control group (normal sleep condition). The reaction time of the SD group was significantly longer than that of the control group for all emotional categories. The intensity rating of facial emotions showed no difference between the two groups.

**Conclusions:**

Sleep deprivation slows individuals’ reactions in facial emotion recognition tasks and weakens their ability to recognize sadness and happiness. Future studies should identify the effects of SD, SD duration, and recovery time on different types of emotion recognition.

## Introduction

Sleep deprivation (SD) is a condition in which a person is unable to obtain enough sleep. The National Sleep Foundation recommends that adults sleep 7–9 hours every night. However, the American Thoracic Society reported that approximately 35% of adults sleep less than seven hours during a typical 24 hours (Bandyopadhyay & Sigua, 2019). Sleep deprivation can be divided into i) acute SD, referring to wake periods that last beyond 16–18 hours (usually lasting one or two days) (Cirelli et al., 2023) and ii) chronic SD, an insufficient sleep syndrome defined by the American Academy of Sleep Medicine as curtailed sleep that persists for three months or longer (Sateia, 2014) or chronic sleep deficiency or insufficient sleep, manifested as sleep fragmentation or other disruptions caused by ongoing SD or poor sleep (Suni, 2022). The causes of SD are complex, and the common reasons for SD in adults are poor sleeping habits, circadian rhythm disturbances, sleep disorders, and other medicinal or dietary factors (Bandyopadhyay & Sigua, 2019). Sleep deprivation alters prefrontal and parietal cortical activity (Krause et al., 2017), which are also involved in emotional processing (Goel et al., 2009). Hence, SD-induced disturbances in cortical activity may lead to specific alterations in individuals’ cognitive and emotional behaviors (Krause et al., 2017).

The effects of SD on emotions are manifold. First, insufficient sleep may affect accurate recognition of sensory (facial, visual, or auditory) stimuli. For example, the accuracy of the facial emotion identification task during a night of partial sleep restriction can be significantly reduced (Nasrini et al., 2020). Decreased activity in the superior parietal lobule and right intraparietal sulcus may be related to SD-induced visuospatial perception, memory, and reasoning impairments (Javaheripour et al., 2019). Second, SD can alter subjective emotional experiences, increasing irritability and affective volatility (Horne, 1985), and has a moderate effect on increasing negative emotions and a large effect on decreasing positive emotions (Tomaso, Johnson & Nelson, 2021). Third, SD changes how individuals understand emotions, impairing emotional regulation (Palmer & Alfano, 2017) and cognitive control (such as attention and memory) of emotions (Alhola & Polo-Kantola, 2007; Almondes, Júnior & Alves, 2016; Tempesta et al., 2018). This result may amplify the negative emotional consequences of disruptive daytime events while blunting the positive benefits associated with rewarding or goal-enhancing activities (Zohar et al., 2005). Finally, sleep conditions are closely related to psychological health (Li et al., 2016). Inadequate sleep is a common factor influencing psychiatric disorders such as anxiety and mood disorders (Palmer & Alfano, 2017). For example, Pires et al.’s (2016) review found that SD significantly increased an individual’s anxiety state.

The effect of sleep disturbances on facial emotional recognition—which is a component of emotional response that includes emotion category identification and reaction time (RT)—has been investigated in many studies (Kyle et al., 2014; Nasrini et al., 2020; van der Helm, Gujar & Walker, 2010). Lack of sleep significantly influences an individual’s reaction to different emotions, which may impair their ability to accurately discriminate between threat and safety signals (Palmer & Alfano, 2017; Tempesta et al., 2018). Sleep deprivation may impair the viscerosensory regions of the anterior insula, anterior cingulate cortices, and subcortical amygdala activity (Goldstein-Piekarski et al., 2015; Krause et al., 2017). For example, sleep loss leads to a generalized, nonspecific increase in amygdala activity in response to aversive and neutral emotional visual stimuli, resulting in a shift of the dynamic spectrum of emotion recognition in facial cues to the direction of negative emotion (Krause et al., 2017).

Although many studies have investigated the influence of SD on facial emotion recognition, the results of different emotion categories vary. In terms of facial emotion recognition accuracy, Kyle et al. (2014) showed no difference in the accuracy of recognizing emotions in participants with or without insomnia, which is similar to Brand et al. (2018). However, several studies have shown that the accuracy of recognizing happiness and sadness differed between participants with and without SD (Crönlein et al., 2016; Killgore, 2017). Regarding RT of facial emotion recognition, Cote’s study showed a significant difference between the SD and control groups, but null results were found in other studies (Holding et al., 2017; Almondes et al., 2020). Ratings of emotional intensity also vary among different emotions between the SD and control groups (Akram, 2020; Kyle et al., 2014; van der Helm et al., 2010). Almondes et al. (2016) summarized the relationship between SD and emotion recognition and the method of facial recognition tasks of the included studies and showed that SD led participants take longer to respond and have lower accuracy in emotion recognition. However, the effect size of the decline in specific emotional category recognition in individuals with SD has never been evaluated through a meta-analysis. Therefore, the purpose of this study is to identify the effect of SD on emotion recognition regarding three aspects (accuracy of recognition, RT, and intensity of emotion recognition) and to determine its effect size.

## Method

### Research Strategy

This systematic review was conducted according to the Preferred Reporting Items for Systematic Reviews and Meta-Analyses (PRISMA) (Mjp et al., 2021). The registration number in PROSPERO is CRD42021284929. A systematic literature search was performed on PubMed, Web of Science, Embase, PsychINFO, MEDLINE, and China National Knowledge Infrastructure for English and Chinese languages within their complete timespans until November 2022. Search terms included (‘Sleep Disorders, Intrinsic’) OR (‘Sleep Initiation and Maintenance Disorders’) OR (Dyssomnias) OR (insomnia* OR hyposomnia OR ‘sleep disorders’ OR sleepless OR anypnia OR agrypnia OR somnipathy OR ‘sleep depriv*’)) AND (‘emotional function’ OR ‘emotional accuracy’ OR ‘emotion recognition’ OR ‘emotion perception’ OR ‘emotion identification’ OR ‘emotion discrimination’ OR ‘emotion differentiation’ OR ‘emotion integration’ OR ‘emotional processing’ OR ‘affective function’ OR ‘affect recognition’ OR ‘affect perception’ OR ‘affect discrimination’ OR ‘affect identification’ OR ‘affect integration’ OR ‘affective processing’ OR ‘facial emotion recognition’). The reference lists of the retrieved articles were also manually searched. All identified studies were collated and uploaded into EndNote X7, and duplicates were checked and removed.

### Study Eligibility

Articles were screened according to the following inclusion criteria: (1) participants aged 18 years or older; (2) acute SD or diagnosed with insomnia using professional diagnostic tools such as the International Classification of Sleep Disorders, the Diagnostic and Statistical Manual of Mental Disorders, Fifth Edition, or other sleep criteria (total sleep time [TST]; sleep onset latency [SOL]; wake after sleep onset [WASO]; sleep efficiency [SE]) monitored by professional equipment considered insomnia; (3) control group: normal sleep condition; (4) at least one emotional recognition task; and (5) sufficient information to calculate mean differences. Articles were excluded if they were (1) animal studies; (2) reviews, meta-analyses, editorials, or conference abstracts; and (3) data from the same participants reported in previous articles. Two independent reviewers (Li MY and Ma CF) screened titles and abstracts to assess eligibility according to the inclusion and exclusion criteria.

### Data Extraction

Two authors (Li MY and Ma CF) independently assessed the eligibility of the records. The third author (Wu C) resolved disagreements between the two authors. The studies’ basic characteristics (i.e., first author’s name, publication time, country of study, sample size, age, sex, emotional recognition task content, and relevant data), means, standard deviations, and sample sizes were extracted for both the SD and control groups. The corresponding author was contacted if the data in the text or tables were incomplete. If there were no responses, the data were measured or estimated according to the figures (mean and standard deviation or standard error) in articles by two researchers to ensure the consistency of the measures. The data of different types of stimuli material (such as Cote’s study (2014) contains full or morphed faces) were calculated as an average value.

### Quality Appraisal of Selected Studies

We used the JBI Critical Appraisal Checklist for Quasi-Experimental Studies (JBICAC for Q-Es) to assess the methodological quality of the studies (Moola et al., 2020; Tufanaru et al., 2020). The appraisal process was conducted by two independent reviewers (Li MY and Li RY) who had completed evidence-based nursing training, and any differences in opinion were discussed with a third researcher (Wu C).

### Statistical Analysis

Meta-analysis was performed using Review Manager software. (Review Manager (RevMan) [computer program] Version 5.4. Copenhagen: The Nordic Cochrane Centre, The Cochrane Collaboration). The effect size was summarized as the mean difference (MD) and 95% confidence interval (95% CI). Heterogeneity of the included studies was estimated using I^2^ statistics (Higgins et al., 2003). The fixed-effects model was used when I^2^ was < 50%, indicating low heterogeneity. The random-effects model was used when I^2^ was ≥ 50% (moderate heterogeneity) and I^2^ ≥ 75% (high heterogeneity). Leave-one-out sensitivity analyses and meta-regression were conducted to detect sources of heterogeneity. Contour-enhanced funnel plots and Egger’s tests were used to examine the possibility of publication bias.

## Results

### Study Selection

**Figure 1:**
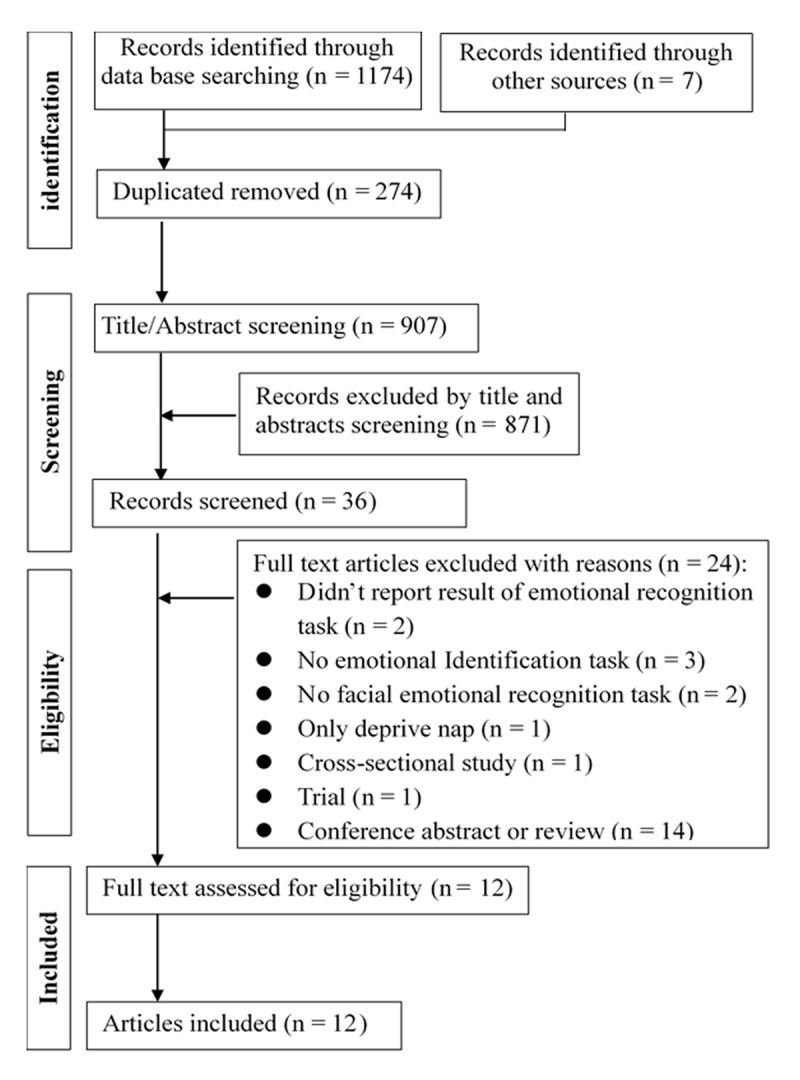
Flow chart of study selection.

A total of 1181 studies were identified. After removing 274 duplicate articles and excluding 871 articles by screening for titles and abstracts, 36 full texts were read, and 24 articles were further excluded for not meeting the inclusion criteria (see Figure 1). Finally, 12 studies were included in the systematic review and meta-analysis. Figure 1 shows the details of the study selection process. The 12 studies included 414 participants in the SD group and 399 in the control group. All participants’ mean age (reported in 10 studies) ranged from 19.7 to 51.6 years. Table 1 presents the characteristics of the included studies.

**Table 1:** Characteristics of Included Studies.


STUDY	COUNTRY	STUDY TYPE	SD GROUP				C GROUP			EMOTIONAL RECOGNITION TASK					
		
			NUMBER (MALE)	AGE	INSOMNIA CRITERIA	SD TIME	NUMBER (MALE)	AGE	SLEEP CONDITION	STIMULI MATERIAL	DURATION OF STIMULI	STIMULI MATERIAL NUMBER	NUMBER OF LABEL CHOICES	EMOTIONAL CATEGORIES	OUTCOMES

Almondes et al. (2020)	①	Q-ES	11 (4)	31.3 ± 9.4	DSM-5	–	15 (5)	24.8 ± 4.6	without sleep disorder	NimStim Set, static/dynamic faces	1, 2, 3, and 4s	Task1: 64; Task2: 64; Task3: 72	4	Ha, Ang, Fe, Sa, B	Accuracy, RT, neutral emotion attribution

Holding et al. (2017)	②	Q-ES	90	–	–	24 h	91	–	from 11:00 p.m. ± 1h to 7:00 a.m. ± 1h	GEMEP, audio/visual/audio-visual	1 ~ 5s	72	12	Anx, De, Di, Ang, Ha, Sa, Fe, In, Ir, Pl, Pr, Re	Accuracy, RT

Kyle et al. (2014)	③	Q-ES	15 (10)	47.1 ± 10.5	SOL/WASO > 30 min, TST ≤ 6 h, SE < 85%	–	16 (10)	47.1 ± 10.8	SOL and WASO < 15 min, TST > 6 h, SE > 85%, Sleep from 10:00 p.m. to 08:00 a.m.	Ekman, static faces	until reaction	80	4	Ang, Fe, Ha, Sa	Accuracy, intensity^#^

Maccari et al. (2014)	④	Q-ES^*^	18 (3)	24.28 ± 2.30	–	24 h	18 (3)	24.28 ± 2.30	from 11:30 p.m. ± 1h to 7:30 a.m. ± 1h	static faces and words	150ms	Task1: 18; Task2: 12	3	A, B, C	Accuracy, RT

Crönlein et al. (2016)	⑤	Q-ES	25 (2)	51.6 ± 10.9	ICSD	–	24 (9)	45.3 ± 8.8	PSQI ≤ 6	Ekman, static faces	300ms	42	6	Ang, Anx, Fe, Ha, Di, Sa	Accuracy

Akram et al. (2020)	③	Q-ES	63 (9)	20.32 ± 4.08	clinically insomnia symptoms, ISI ≥ 15	–	56 (15)	19.69 ± 4.07	ISI < 5	KDEF, static faces	until reaction	12	rating 1~96	B	Intensity rate

van der Helm et al. (2010)	⑥	CCT	20 (10)	–		24 h	17 (9)	–	sleep 7.9 ± 1.6 h at night	Ekman, static faces	≤ 2s	24	4	Sa, Ang, Ha, B	Intensity

Killgore et al. (2017)	⑥	Q-ES^*^	54 (29)	23.5 ± 4.0	–	24 h	54 (29)	23.5 ± 4.0	from 8:00 p.m. to 8:00 a.m.	Ekman, static faces	≤ 5s	120	6	Ha, Sa, Ang, Di, Fe, Su	Accuracy

Sack et al. (2018)	⑤	Q-ES	40	–	–	≥ 20 h	50	–	normal night sleep	facial emotional video	2, 4, 6, 8, 10s	100	4	Ang, Di, Fe, Sa	Accuracy, RT

Stenson et al. (2021)	⑥	CCT	40	–	–	24 h	20	–	from 10:00 p.m. to 8.00 a.m.	NimStim Set, static faces	1.5s	165	rating –2 ~ 2	A, B, C	Accuracy, RT, intensity^#^

Cote et al. (2014)	⑦	CCT	24 (13)	–	–	24 h	24 (13)	–	from 11:00 p.m. to 7:00 p.m.	NimStim Set, FF and MF	500ms	FF: 360 MF: 576	4	Ha, Sa, Ang, Fe	Accuracy, RT

Huck et al. (2008)	⑥	Q-ES^*^	14	–	–	24 h, 48 h	14	–	8 hours in bed	Ekman, sample/blend facial task	≤ 5s	20	6	Ang, Su, Fe, Sa, Ha, Di	Accuracy


* Before-after study; ^#^ The task of intensity rating was performed after choosing the emotional category, and the stimulus disappeared at the same time. **Emotional categories:** A, positive; B, neutral; C, negative; Ha, happiness; Sa, sadness; Ang, anger; Fe, fear; Di, disgust; Anx, anxiety; Su, surprise; In, interest; Ir, irritation; Pl, pleasure; Pr, pride; Re, relief; De, despair. **Abbreviation:** SD, sleep deprivation; C group, control group; Q-ES, quasi-experimental study; CCT, controlled clinical trial; ISI, Insomnia Severity Index; PSQI, Pittsburgh Sleep Quality Index; DSM-5, The Diagnostic and Statistical Manual of Mental Disorders-5th edition; TST, total sleep time; SOL, sleep onset latency; WASO, wake after sleep onset; ICSD, the International Classification of Sleep Disorders; SE, sleep efficiency; RT, reaction time; GEMEP, Geneva Multimodal Emotion Portrayal; KDEF, Karolinska Directed Emotional Faces database; IAPS, International Affective Picture System; FF, Full face task; MF, Morphed face task. **Country:** ①Brazil; ②Sweden; ③England; ④Spain; ⑤German; ⑥America; ⑦Canada; ⑧Italy.

### Quality Assessment

We used the JBICAC for Q-Es to assess the quality of the studies. Three studies (Cote et al., 2014; Stenson et al., 2021; van der Helm et al., 2010) were assessed as Grade A, and nine studies (Akram, 2020; Almondes et al., 2020; Crönlein et al., 2016; Holding et al., 2017; Huck et al., 2008; Killgore, 2017; Kyle et al., 2014; Maccari et al., 2014; Sack, Broer, & Anders, 2018) were assessed as Grade B (Table 2).

**Table 2:** Quality Assessment of Selected Studies.


STUDY	1	2	3	4	5	6	7	8	9	GRADE

Almondes et al. (2020)	Y	Y	U	Y	N	Y	Y	Y	Y	B

Holding et al. (2019)	Y	Y	Y	Y	N	Y	Y	Y	Y	B

Kyle et al. (2014)	Y	Y	Y	Y	N	Y	Y	Y	Y	B

Maccari et al. (2014)	Y	NA	NA	N	Y	Y	Y	Y	Y	B

Crönlein et al. (2016)	Y	Y	Y	Y	N	Y	Y	Y	Y	B

Akram et al. (2020)	Y	Y	Y	Y	N	Y	Y	Y	Y	B

van der Helm et al. (2010)	Y	Y	Y	Y	N	Y	Y	Y	Y	A

Killgore et al. (2017)	Y	NA	NA	N	Y	Y	Y	Y	Y	B

Sack et al. (2018)	Y	Y	Y	Y	N	Y	Y	Y	Y	B

Stenson et al. (2021)	Y	Y	Y	Y	Y	Y	Y	Y	Y	A

Cote et al. (2014)	Y	Y	Y	Y	Y	Y	Y	Y	Y	A

Huck et al. (2008)	Y	NA	NA	N	Y	Y	Y	Y	Y	B


Capital letters: Y: Yes; N: No; U: Unclear; NA: Not applicable. A: All of the items were assessed as “Yes”; B: part of the items was not assessed as “Yes”; C: none of the items were assessed as “Yes.”

### Emotion Recognition Accuracy

**Figure 2:**
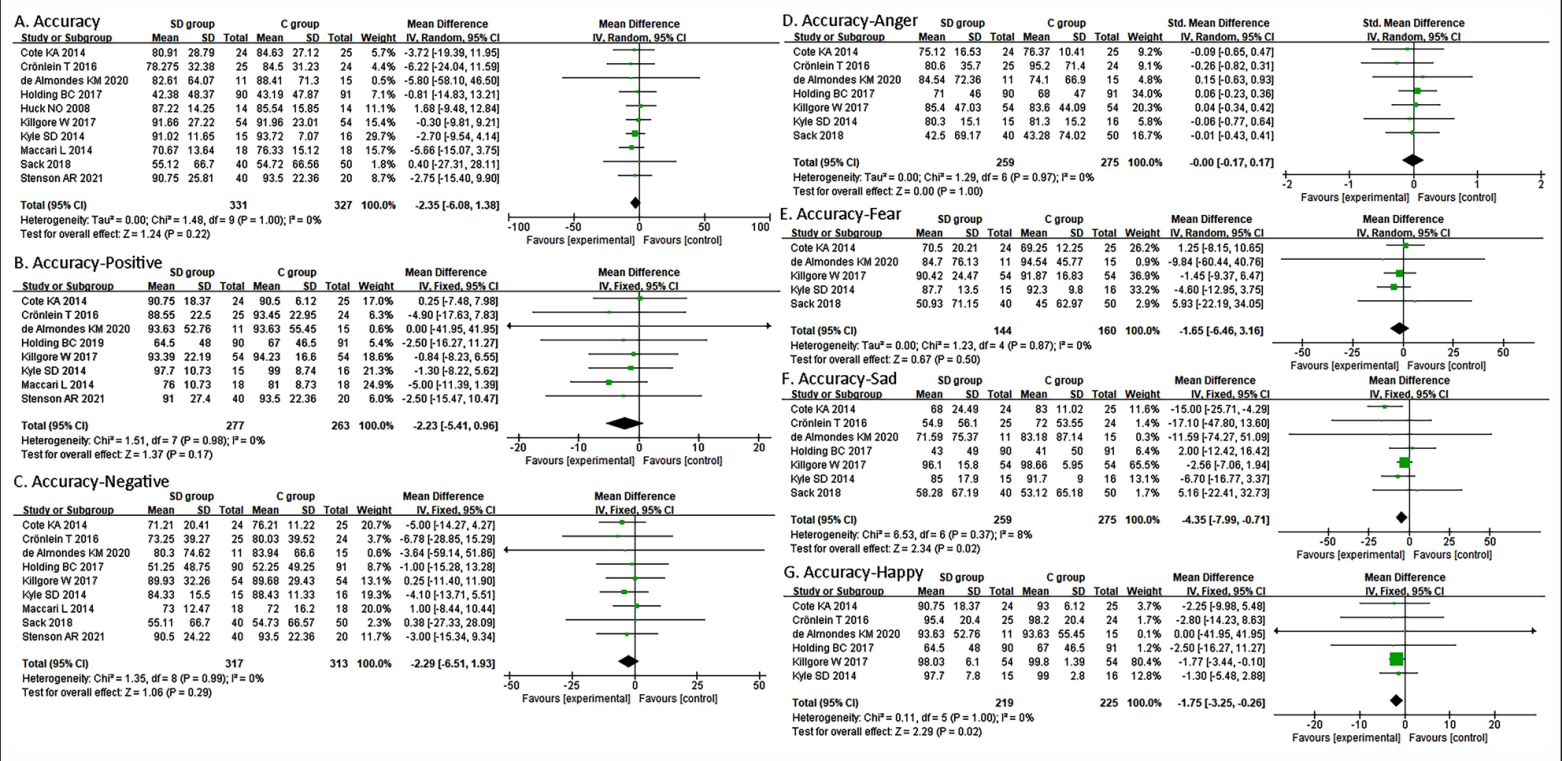
Forest plot of comparison of emotion recognition accuracy.

Ten studies (Almondes et al., 2020; Cote et al., 2014; Crönlein et al., 2016; Holding et al., 2017; Huck et al., 2008; Killgore, 2017; Kyle et al., 2014; Maccari et al., 2014; Sack et al., 2018; Stenson et al., 2021) evaluated the accuracy of emotion category recognition. The results of the fixed-effects model showed no significant difference in accuracy across emotion categories between the SD and control groups (MD = –2.35, 95% CI, –6.08 to 1.38, I2 = 0%; Figure 2A). Eight studies (Cote et al., 2014; Crönlein et al., 2016; Holding et al., 2017; Killgore, 2017; Kyle et al., 2014; Maccari et al., 2014; Almondes et al., 2020; Stenson et al., 2021) evaluated positive emotions (happiness, joy, pleasure, and pride). The results showed no difference in positive emotion recognition between SD and control participants (MD = –2.23; 95% CI, –5.41 to 0.96; I2 = 0%; Figure 2B). Nine studies evaluated negative emotions (fear, sadness, disgust, and anger) (Cote et al., 2014; Crönlein et al., 2016; Holding et al., 2017; Killgore, 2017; Kyle et al., 2014; Maccari et al., 2014; Almondes et al., 2020; Sack et al., 2018; Stenson et al., 2021). The results of the fixed-effects model showed no significant difference in recognition accuracy between the SD and control groups across the negative emotion categories (MD = –2.29, 95% CI, –6.51 to 1.93, I^2^ = 0%; Figure 2C). The results for overall, positive, and negative accuracy remained insignificant after the leave-one-out sensitivity analyses. Seven studies (Cote et al., 2014; Crönlein et al., 2016; Holding et al., 2017; Killgore, 2017; Kyle et al., 2014; Almondes et al., 2020; Sack et al., 2018) reported a single emotion category recognition task. All the seven studies evaluated anger and sadness; six studies evaluated happiness (Cote et al., 2014; Crönlein et al., 2016; Holding et al., 2017; Killgore, 2017; Kyle et al., 2014; Almondes et al., 2020); five studies evaluated fear (Cote et al., 2014; Killgore, 2017; Kyle et al., 2014; Almondes et al., 2020; Sack et al., 2018); three studies evaluated disgust (Crönlein et al., 2016; Holding et al., 2017); and two studies evaluated anxiety (Crönlein et al., 2016; Killgore, 2017; Holding et al., 2017) and surprise (Crönlein et al., 2016; Killgore, 2017). As seen in Figure 2 D–G, there was no difference in fear and anger recognition accuracy between the SD and control groups (anger: MD = –0.00; 95% CI, –0.17 to 0.17; I2 = 0%; fear: MD = –1.65; 95% CI, –6.46 to 3.16; I2 = 0%), whereas the SD group performed poorer than the control group in recognizing sadness and happiness (sadness: MD = –4.35; 95% CI, –7.99 to –0.71; I^2^ = 0%; happiness: MD = –1.75; 95% CI, –3.25 to –0.26; I^2^ = 0%). The statistical significance of anger, fear, and sadness did not change after the leave-one-out sensitivity analyses, but happiness became statistically insignificant when Killgore et al.’s (2017) study was removed (MD = –1.68; 95% CI, –5.06 to 1.70; I^2^ = 0%).

One study (Sack et al., 2018) analyzed the effect of stimulus length on accuracy. The results showed that the length of the video had a significant effect on total accuracy (*F* = 8.8, *p* < 0.001), the total accuracy being higher with the longer stimulus (8–10s) than with the shorter stimulus (2–4s). There was also an interaction effect between sleep condition and stimulus length; the total accuracy was significantly higher in the SD group when using the 8–10s stimulus but the difference was not significant when using the 2–4s stimulus (control group, 2–4 s videos, *T* = – 0.02, *p* > 0.200; 8–10 s videos, *T* = 2.5, *p* = 0.008). When recognizing the same length facial emotional video among single category emotions (anger, disgust, fear, and sadness), the difference was also not significant between the different sleep conditions.

Subgroup analyses based on the type of negative emotion recognition task (static and dynamic emotion) showed that the difference in accuracy between the SD group and control group was neither significant in static nor dynamic emotions (static: MD= –2.51, 95% CI, –6.99 to 1.96, I^2^ = 0%; dynamic: MD = 0.50, 95% CI, –24.02 to 25.01, I^2^ = 0%), and there was no significant subgroup difference.

### Emotion Reaction Time

**Figure 3:**
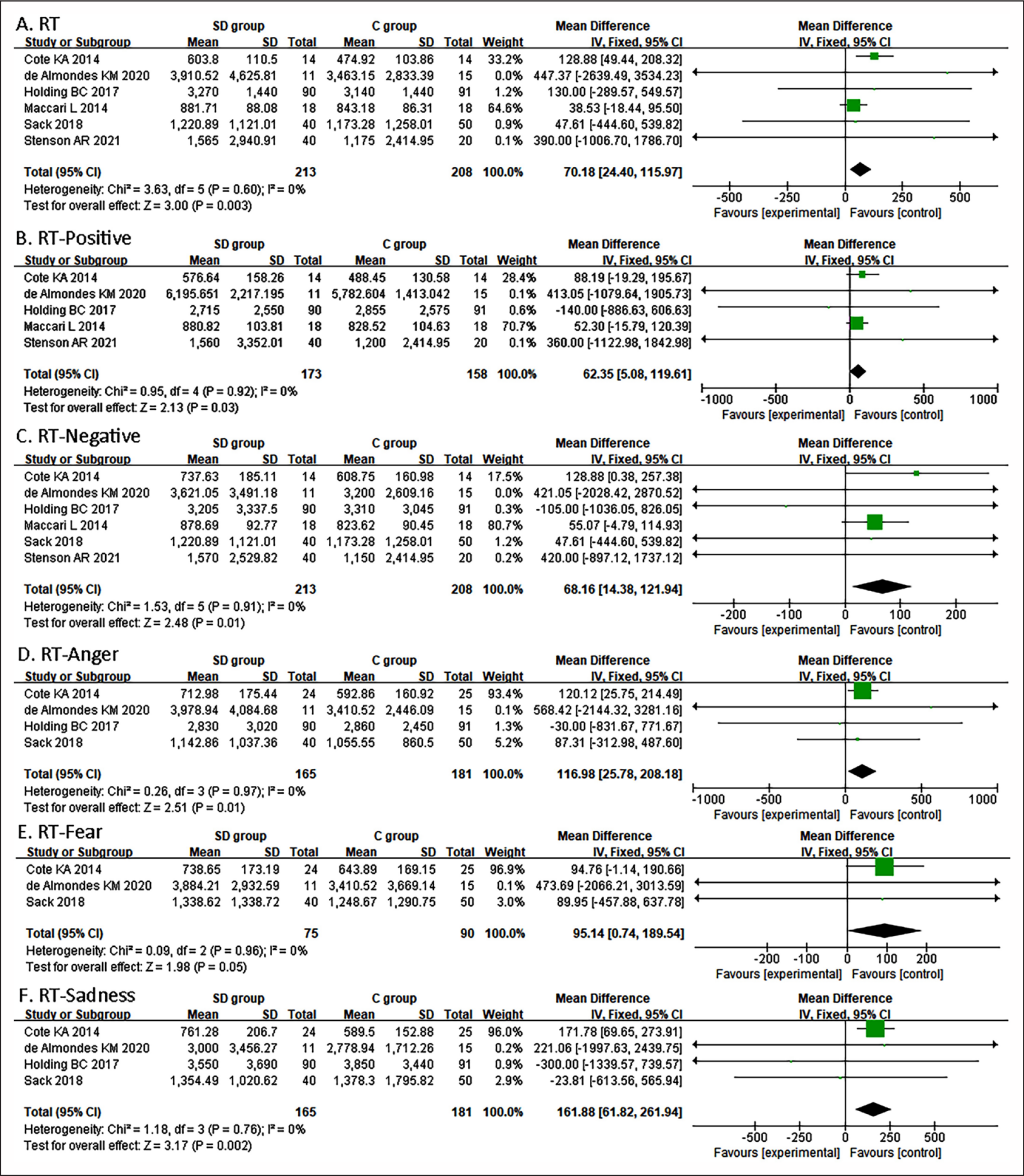
Forest plot of comparison of emotion reaction time.

Six studies (Almondes et al., 2020; Cote et al., 2014; Holding et al., 2017; Maccari et al., 2014; Sack et al., 2018; Stenson et al., 2021) reported RT for recognizing emotions, and one study (Sack et al., 2018) reported only the average RT across emotion recognition. The results of a fixed-effects model showed that the SD group required longer RT for recognizing emotions than the control group (MD = 70.18, 95% CI, 24.40 to 115.97, I^2^ = 0%; Figure 3A). Five studies evaluated positive emotions (Almondes et al., 2020; Cote et al., 2014; Holding et al., 2017; Maccari et al., 2014; Stenson et al., 2021), and six studies evaluated negative emotions (Almondes et al., 2020; Cote et al., 2014; Holding et al., 2017; Maccari et al., 2014; Sack et al., 2018; Stenson et al., 2021). The SD group reacted significantly longer to positive (MD = 62.35; 95% CI, 5.08 to 119.61; I^2^ = 0%; Figure 3B) and negative emotions (MD = 68.16; 95% CI, 14.38 to 121.94; I^2^ = 0%; Figure 3C) than the control group. Sensitivity analyses revealed that the effect size became not significant for the total (MD = 45.26; 95% CI, –10.12 to 100.64), positive (MD = 52.57; 95% CI, –15.57 to 119.77), and negative (MD = 55.27; 95% CI, –3.95 to 114.49) emotion RT when excluding Cote et al.’s (2014) study.

Four studies (Almondes et al., 2020; Cote et al., 2014; Holding et al., 2017; Sack et al., 2018) reported RT for single emotion recognition. As seen in Figure 3 D–F, the RT of anger (MD = 116.98, 95% CI, 25.78 to 208.18; I^2^ = 0%), fear (MD = 95.14, 95% CI, 0.74 to 189.54; I^2^ = 0%;), and sadness (MD = 161.88, 95% CI, 61.82 to 261.94; I^2^ = 0%) of the SD group was significantly longer than that of the control group. Sensitivity analyses revealed that the effect became insignificant for those three single emotions when excluding Cote et al.’s (2014) study (anger: MD = 45.26, 95% CI, –282.51 to 427.59; fear: MD = 55.27, 95% CI, –428.50 to 642.52; sadness: MD = –75.22, 95% CI, –574.99 to 424.56).

### Emotional Intensity

**Figure 4:**
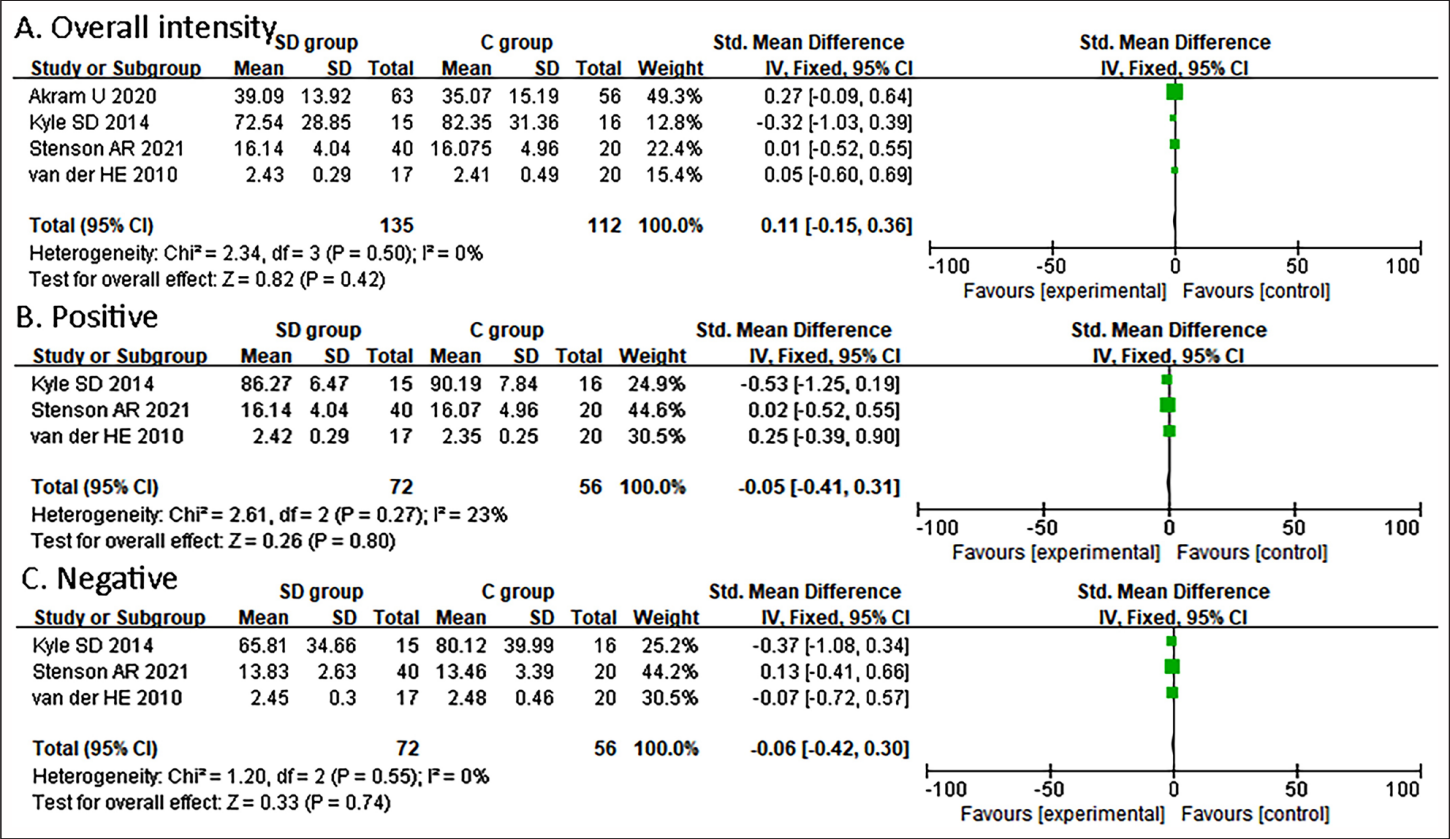
Forest plot of comparison of emotional intensity.

Four studies (Akram, 2020; Kyle et al., 2014; Stenson et al., 2021; van der Helm et al., 2010) reported the results of recognition of emotional intensity. One study (Akram, 2020) reported the intensity recognition of neutral emotions, and the other three studies reported the intensity rating of anger (Kyle et al., 2014; Stenson et al., 2021; van der Helm et al., 2010), fear (Kyle et al., 2014), happiness (Akram, 2020; Kyle et al., 2014; van der Helm et al., 2010), and sadness (Akram, 2020; Kyle et al., 2014; van der Helm et al., 2010). No significant difference was observed between the two groups in intensity rating across emotion categories (0.11; 95% CI, –0.15 to 0.36, I^2^ = 0%; Figure 4A). The difference was not significant in positive intensity rating (SMD = –0.05; 95% CI, –0.41 to 0.31, I^2^ = 23%, Figure 4B). The difference in negative intensity was also not significant between the two groups (SMD = –0.06; 95% CI, –0.42 to 0.30, I^2^ = 0%, Figure 4C).

Evidence suggests a sex difference in insomnia and depression, with female participants being particularly sensitive to the interaction of mood disorders and sleep abnormalities (Salk, Hyde & Abramson, 2017; Suh, Cho & Zhang, 2018). A study by van der Helm et al. (2010) explored gender differences in the effect of SD on emotional intensity ratings and found that the intensity rating of female participants in the SD group was significantly lower in the recognition of anger emotions (MD = –0.22; 95% CI, –0.40 to –0.04), and there were no differences in male participants (anger: MD = –0.04; 95% CI, –0.45 to 0.37). Intensity ratings of happiness and sadness were not significant in either female or male participants (happiness: female: MD = 0.15; 95% CI, –0.09 to 0.39, male: MD = –0.18; 95% CI, –0.51 to 0.15; sadness: female: MD = –0.03; 95% CI, –0.26 to 0.20, male: MD = –0.10; 95% CI, –0.54 to 0.34).

### Meta Regression

The number of label choices may influence the participant’s accuracy in reacting to emotions; hence, we added this variable to the meta-regression model. The number of label choices did not significantly influence the meta-analysis results (SE = 0.87, 95%CI: [–1.17, 2.24], *p* > 0.1). Meta-regression showed that the number of label choices did not significantly influence the results of RT (SE = 24.43, 95%CI: [–32.75, 62.99], *p* > 0.1).

### Publication Bias

**Figure 5:**
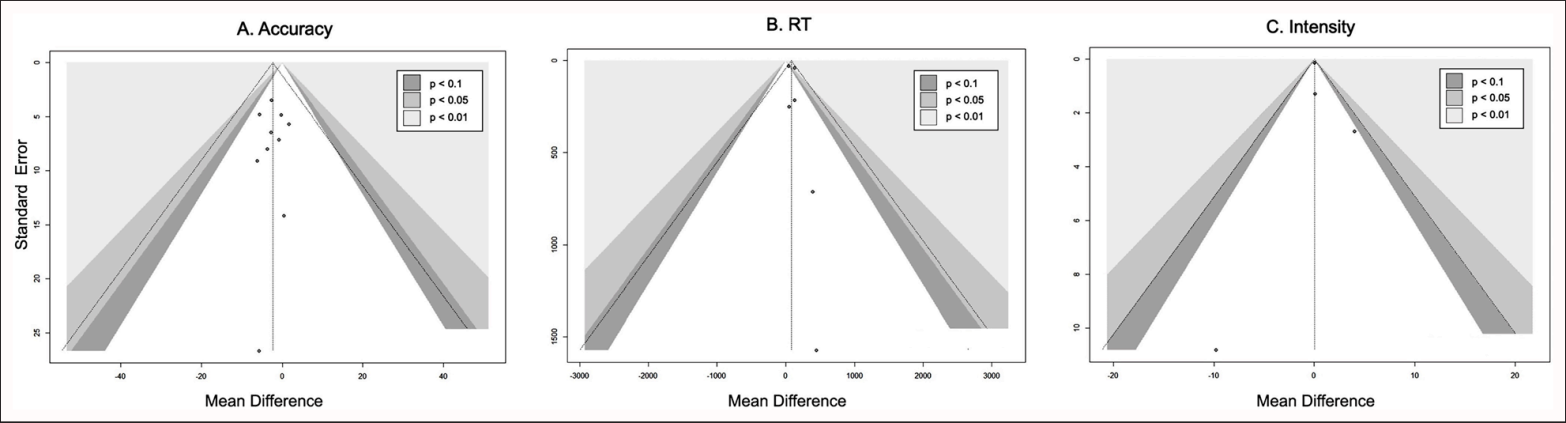
Funnel plot of the emotion recognition accuracy, emotion reaction time, and emotional intensity.

Visual inspection of the funnel plot showed near symmetry (Figure 5A) for the total emotional recognition accuracy. Egger’s test identified no significant publication bias (*t* = –0.07, 95%CI: –2.22 to 1.98, *p* = 0.95). The contour-enhanced funnel plots of RT and intensity rating showed that almost all studies lay in the regions of *p* > 0.1, and the distribution of studies was almost symmetrical (Figure 5B, C), indicating a low possibility of publication bias.

### Summary of Findings

Table 3 shows the findings of primary outcomes assessed according to the GRADE approach (Guyatt et al., 2011). We summarized seven primary outcomes according to the Cochrane guidelines of Completing Summary of Findings tables and grading the certainty of evidence (Higgins & Thomas, 2022). All the outcomes started with low quality because the studies were not randomized control studies, and no outcomes upgrade the certainty of the evidence owing to not finding a large effect, dose-response, or plausible confounding. Hence, all the study outcomes were identified as ‘low quality’ (see Table 3 for details). However, the quality assessment results showed that all the included studies had a sound methodological quality using the JBICAC for quasi-experimental studies (Table 2), which proved the reliability of the evidence to some extent.

**Table 3:** Summary of Findings.


OUTCOMES	PARTICIPANTS (n)		INTERVENTION VS. COMPARATOR MEAN DIFFERENCE (95% CI)	QUALITY OF EVIDENCE

	SD GROUP	CONTROL GROUP		

Total Accuracy	acute SD: 280 insomnia: 51	normal sleep: 327 (10 studies)	–2.35% [–6.08, 1.38] **lower** across the emotion category accuracy	⊕⊕⊖⊖

Positive emotional accuracy	acute SD: 226 insomnia: 51	normal sleep: 263 (10 studies)	2.23% [–5.41, 0.96] **lower** in positive emotion accuracy	⊕⊕⊖⊖

Negative emotional accuracy	acute SD: 266 insomnia: 51	normal sleep: 313 (9 studies)	2.32% [–6.55, 1.90] **lower** in negative emotion accuracy	⊕⊕⊖⊖

Total RT	acute SD: 202 insomnia: 11	normal sleep: 208 (6 studies)	reacting 70.18ms [24.40, 115.97] **longer** across the emotion categories	⊕⊕⊖⊖

Positive emotional RT	acute SD: 162 insomnia: 11	normal sleep: 158 (5 studies)	reacting 62.35ms [5.08, 119.61] **longer** in positive emotions	⊕⊕⊖⊖

Negative emotional RT	acute SD: 202 insomnia: 11	normal sleep: 208 (6 studies)	reacting 68.16ms [14.38, 121.94] **longer** in negative emotions	⊕⊕⊖⊖

Intensity	acute SD: 57 insomnia: 78	normal sleep: 112 (4 studies)	rating 0.11% [–0.15 to 0.36] **lower** across emotions	⊕⊕⊖⊖


**Abbreviation:** SD, sleep deprivation.

## Discussion

The present meta-analysis included 12 studies to examine and compare the accuracy and RT of emotion recognition between the SD and normal sleep groups. Overall, the SD group performed more poorly (i.e., with lower accuracy) on recognizing the sadness emotion, but not on other emotional-category recognition. The SD group’s RT was significantly longer than that of the normal sleep condition across positive and negative emotions. Sleep deprivation did not affect the recognition of facial emotion intensity. The findings indicated that acute SD may not impair the recognition of positive emotions and most negative emotions, except for sadness, but leads to longer processing times for negative emotions.

The results of accuracy and RT proved that SD impairs the perception of emotional expressions (reflected in impaired emotional recognition accuracy) and the speed of processing emotions (reflected in longer RT). We found that SD had a significant effect only on the recognition accuracy of sadness. This may be because, compared to fear and anger, sad faces are relatively less salient or important for one’s survival and well-being. As sadness is characterized by low arousal and negative valence, individuals may need more effort or control over processing when cognitive resources are affected by challenges such as SD. The dorsolateral prefrontal cortex (PFC) is an important brain area involved in distinguishing facial emotions (Yoo et al., 2007a), which is also sensitive to sleep loss (Takeuchi et al., 2018). Sleep deprivation could impair prefrontal connections with subcortical and temporoparietal areas. These connections are important for visual cognition and emotional perception such as the process of facial recognition and perception (Motomura et al., 2014). Another possible neurobiological explanation is that sadness emotions may be processed differently because they are associated with lower autonomic and central nervous system arousal. Sadness recognition may, therefore, be particularly vulnerable during SD because arousal of these systems is profoundly impacted during long-term wakefulness (Cote et al., 2014).

Overall, in the emotional classification task, the response time of the SD participants was longer than that of the control group participants. Specifically, SD participants took longer to recognize negative emotions than the control group, but the difference was not significant for recognizing positive emotions. In this study, participants took longer to recognize negative emotions than positive emotions. This suggests that SD causes individuals to spend more time identifying emotions, especially negative ones. From the perspective of attention, extended SD can cause attentional impairments. Chee et al. (2011) showed that SD can lead to reductions in functional MRI signals in the dorsolateral PFC and intraparietal sulcus, which are related to impaired executive function and attention. Sleep deprivation can decrease task-related activity in the frontal and parietal regions and diminish activity in and connectivity with the extrastriate visual cortex during attention tasks. This may result in deficiencies in attending to specific stimuli. From the perspective of neurobiological mechanisms of RT differences in positive and negative emotions, participants react differently to positive and negative emotions in the amygdala, orbitofrontal cortex, and medial prefrontal cortex. Impairment in the amygdala has a broad adverse impact on recognizing negative valence emotions (including anger, fear, and disgust) (Gallagher & Chiba, 1996), and individual impairment in the orbitofrontal cortex could decrease their ability to recognize fear (Adolphs, 2002). Disrupting processing within the medial prefrontal cortex would make participants spend longer recognizing angry faces (Harmer et al., 2001). Hence, PFC impairment and its connections with other brain regions (including the amygdala) decreases caused by SD may be why the SD group takes longer to respond to negative emotions.

Regarding emotional intensity, we did not find a significant difference between SD groups and control groups, and the effects of different studies cross zero (Cross emotion: –0.15 to 0.36; Positive emotion: –0.41 to 0.31; Negative emotion: –0.42 to 0.30). The results of our meta-analyses did not corroborate (total effect of intensity not significant) the brain mechanism observed in a previous study, which indicates that SD may lead to reduced connectivity with medial and orbito-frontal areas, which would decrease the threshold of emotional reactivity across different affects (Yoo et al., 2007b). However, the results show a tendency in the total effect that the recognition of intensity of SD groups was lower than control groups (Figure 5), verifying the previous mechanism to some extent. Moreover, there may be differences in emotion processing between experimental and real-life environments. Some studies asked participants to complete the selection and rating of each emotional stimulation quickly (Maccari et al., 2014; Stenson et al., 2021), which is different from real social conditions where people always have enough time to ponder the outside world’s emotional stimuli. Moreover, sex differences may exist in the recognition of emotional intensity as seen in one study: female participants in the SD group recognized less intensity of anger compared to the control group, but this difference was not significant in male participants (van der Helm et al., 2010). The possible neurobiological mechanism was that women showed greater homeostatic sleep sensitivity and drive than men, such that small changes in sleep conditions could trigger a stronger sleep rebound in female than male participants. This sensitivity may explain the consistent effect imposed by sleep loss in female participants (van der Helm et al., 2010). However, the mechanisms underlying sex differences in emotional reactivity to different categories of emotions need to be further explored.

### Limitations and Implications

Three types of SD have been identified in previous studies: acute SD (referring to wake periods that last one or two days) (Cirelli et al., 2023), chronic SD (curtailed sleep that persists for three months or longer) (Sateia, 2014), and chronic sleep deficiency or insufficient sleep (sleep fragmentation or other disruptions caused by ongoing SD or poor sleep) (Suni, 2022). In this review, chronic SD was defined as insomnia symptoms that continued for more than one month, and acute SD was defined as continuously depriving sleep for 20 hours at least. Other SD conditions, such as sleep apnea, rapid eye movement SD (such as nap deprivation), sleep restriction, and medicine- or caffeine-caused SD, were not included in this review (Gujar, McDonald, Nishida & Walker, 2011; Huck et al., 2008; Smith et al., 2021). The impact of different types of SD on emotional recognition should be clarified in future studies. Moreover, in those studies of acute passive SD, we extracted only one-day (almost 24 hours) SD data, and a few studies (Huck et al., 2008) reported longer SD times and recovery effects. Therefore, the time effect and resilience of SD on emotional recognition were not examined in this review, which could be a future research direction.

There are also some implications for the development of theories and clinics. Regarding the Gross model of emotional regulation, the process of emotion regulation including situation selection, attentional deployment, cognitive change, and response modulation (McRae & Gross, 2020), Palmer and Alfano (2017) found that sleep loss could influence emotional regulation through the process of attentional deployment for that SD impaired the efficacy of distraction which may decrease individuals’ ability of emotion regulation. This study further found that SD could impair individuals’ attention to negative emotions, leading them to take a longer time to recognize negative emotions. This means that it would harder to distract individuals with sleep loss from negative emotions, which may deteriorate their mood. Hence, developing and using distraction strategies may be an efficient intervention for solving emotional problems in patients with SD. These distraction strategies have been confirmed to be effective in patients with depression and anxiety (Efinger, Thuillard & Dan-Glauser, 2019; Smoski, LaBar & Steffens, 2014).

## Conclusion

Sleep deprivation slows an individual’s reaction to negative emotions and weakens the ability to recognize sadness accurately. The effects of SD on single emotion category recognition are not consistent owing to the diverse SD conditions and emotional recognition tasks. It affects individuals’ emotional states by impairing their ability to distract themselves from negative emotions.

## Data Accessibility Statement

All of the data were extracted from the included studies. It can be obtained from the figures. This meta-analysis was registered at PROSPERO with the registration number CRD42021284929.
